# Multiple Functions of MYB Transcription Factors in Abiotic Stress Responses

**DOI:** 10.3390/ijms22116125

**Published:** 2021-06-07

**Authors:** Xiaopei Wang, Yanli Niu, Yuan Zheng

**Affiliations:** State Key Laboratory of Crop Stress Adaptation and Improvement, School of Life Sciences, Henan University, Kaifeng 475001, China; xiaopeiwang160411@163.com (X.W.); nyl0925@vip.henu.edu.cn (Y.N.)

**Keywords:** MYB, transcription factor, R2R3, abiotic stress responses

## Abstract

Plants face a more volatile environment than other organisms because of their immobility, and they have developed highly efficient mechanisms to adapt to stress conditions. Transcription factors, as an important part of the adaptation process, are activated by different signals and are responsible for the expression of stress-responsive genes. MYB transcription factors, as one of the most widespread transcription factor families in plants, participate in plant development and responses to stresses by combining with MYB *cis*-elements in promoters of target genes. MYB transcription factors have been extensively studied and have proven to be critical in the biosynthesis of secondary metabolites in plants, including anthocyanins, flavonols, and lignin. Multiple studies have now shown that MYB proteins play diverse roles in the responses to abiotic stresses, such as drought, salt, and cold stresses. However, the regulatory mechanism of MYB proteins in abiotic stresses is still not well understood. In this review, we will focus mainly on the function of *Arabidopsis* MYB transcription factors in abiotic stresses, especially how MYB proteins participate in these stress responses. We also pay attention to how the MYB proteins are regulated in these processes at both the transcript and protein levels.

## 1. Introduction

MYB transcription factors represent one of the largest protein families in plants. MYB transcription factors are characterized by highly conserved N-terminal MYB DNA-binding domain (DBD) repeats (Rs). Each repeat contains approximately 52 amino acid residues that fold into three α-helices. The second and third helices can form a helix-turn-helix (HTH) structure. Due to the similarity with the c-Myb protein, these repeats are renamed R1, R2, and R3. In plants, MYB transcription factors comprise one to four DNA-binding repeats. However, the majority of MYB proteins contain two repeats and belong to the R2R3–MYB subfamily. Many plant species encode more than 100 R2R3 MYB proteins. In contrast to the N-terminus, the C-terminal region is more variable and responsible for regulatory function. For example, in *Arabidopsis*, 126 R2R3 MYB proteins can be divided into 23 subgroups based on the conserved region of the C-terminus [1,2].

R2R3–MYB proteins have been found to participate in diverse processes, including primary and secondary metabolism, plant development, and responses to biotic and abiotic stresses [1]. Among these functions, MYB proteins have been particularly well studied as regulators of phenylpropanoid metabolism, such as the biosynthesis of proanthocyanidins (PAs), anthocyanins, flavonols, and lignin in plants. For example, MYB proteins can bind to the bHLH transcription factor and WD40 protein to form the MBW complex and activate the biosynthesis of PAs and anthocyanins [3,4,5,6]. Multiple studies have indicated the critical roles of MYB transcription factors in plant stress responses, especially in abiotic stresses [7,8,9]. In this review, we will describe the function of *Arabidopsis* R2R3 MYB proteins in abiotic stress responses, focusing mainly on their roles in drought, high salinity, cold, and heat tolerance. Furthermore, we will discuss how MYB proteins are regulated at the posttranslational level, based on current studies.

## 2. Dual Roles of MYBs in Both UV-B Tolerance and Signaling

Ultraviolet B (UV-B) light is an intrinsic component of sunlight that markedly affects plant development. Exposure of plants to high UV-B radiation induces stress responses such as inhibition of photosynthesis and damage to DNA and other molecules. Plants produce hydroxycinnamic acid derivatives (sinapate esters) and flavonoids to absorb UV-B, thus protecting plants against the harmful effects of UV radiation [10]. *Arabidopsis* MYB4 is well known as a key regulator in UV tolerance for its negative role in UV sunscreen biosynthesis. MYB4 directly represses the transcription of the *C4H* (*cinnamate-4-hydroxylase*) gene, the key enzyme for the biosynthesis of UV sunscreen hydroxycinnamate ester. UV-B light stimulates the expression of *C4H* by negatively regulating the expression of *MYB4*. The MYB4 loss-of-function mutant showed UV-B tolerance due to an increased accumulation level of hydroxycinnamate esters, while overexpression of MYB4 caused a reduced level of UV-B-absorbing compounds, resulting in UV-B hypersensitivity [11]. The expression and localization of MYB4 are precisely controlled. MYB4 can repress *MYB4* expression by binding to its own promoter. While the importin β-like protein, SAD2, interacts with MYB4 via the conserved GY/FDFLGL motif to facilitate nuclear transport of MYB4 for regulation of *C4H* expression. In *sad2* mutant, more MYB4 localized in cytoplasm [12,13]. MYB7, the closest MYB4 homolog, is also involved in the repression of flavonol biosynthesis. Several genes involved in flavonol biosynthesis are upregulated, and the corresponding compounds increase in *myb7* mutant plants [14].

Meanwhile, low-dosage UV-B irradiation elicits a variety of photomorphogenic developments, including inhibition of hypocotyl elongation, root growth, and induction of cotyledon expansion [15,16]. In *Arabidopsis*, the UV-B signal is perceived by the photoreceptor UVR8 (UV RESISTANCE LOCUS 8) [17]. UVR8 forms homodimers in the cytoplasm, while UV-B irradiation induces the monomerization and nuclear accumulation of UVR8 [18]. UVR8 mediates the repression of lateral root growth under UV-B treatment by downregulating the expression of diverse auxin-responsive genes. The function of MYB transcription factors in UV signaling seems to be mediated by the interaction with UVR8. MYB73 and MYB77 interact with UVR8 in a UV-B-dependent manner and act downstream of UVR8 to regulate lateral root growth. When grown in the presence of a high concentration of auxin under UV-B light, the *Arabidopsis myb73 myb77* double mutant exhibited longer lateral roots than the wild type. MYB73 and MYB77 positively regulate the expression of auxin-responsive genes by directly binding to their promoters. The interaction of UVR8 with MYB73 and MYB77 can block their DNA-binding ability and consequently repress the expression of downstream auxin-responsive genes [19]. MYB13 positively regulates UV-B-induced cotyledon expansion and stress acclimation by directly regulating the expression of auxin-responsive and flavonoid biosynthetic genes. MYB13 is predominantly expressed in cotyledons and can be induced by UV-B. UV-B-induced cotyledon expansion was abolished in *myb13* mutants, and *myb13* mutants showed hypersensitivity to UV-B stress, similarly to *uvr8* mutant. UVR8 can interact with MYB13 in a UV-B-dependent manner and differentially modulate the affinity of MYB13 with its targets [20].

## 3. Multifunctional Roles of MYBs in Drought Stress Response

**Figure 1:**
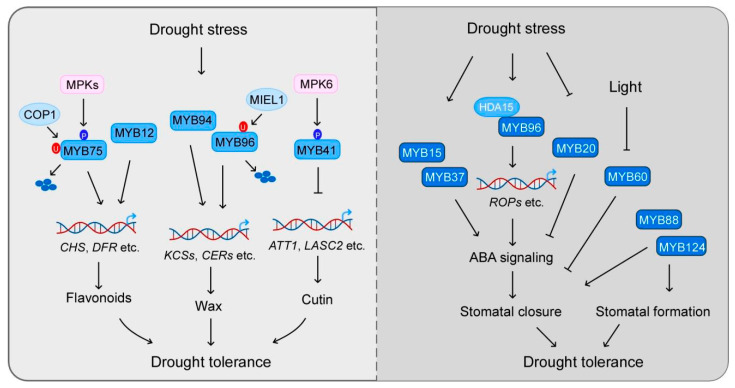
The pivotal role of MYB transcription factors in drought tolerance. In response to drought stress, MYB transcription factors regulate the expression of biosynthetic genes of metabolites, such as flavonoids, wax, and cutin, to modulate plant drought tolerance. Additively, MYB transcription factors participate in stomatal movement, mainly through ABA signaling. MYB60 specially regulates the stomatal aperture in response to light while MYB124/FLP and MYB88 also play critical roles in stomatal development. COP1: constitutively photomorphogenic1; MIEL1: MYB30-interacting E3 ligase 1; ROP: RHO GTPase of plants.

Drought is one of the most important environmental stresses affecting plant growth and crop yield. MYB transcription factors participate in plant drought tolerance through their diverse functions (Figure 1).

### 3.1. Flavonoid and Cuticle Biosynthesis with Drought Tolerance

First, MYB proteins, as one of the most important transcription factor families in plant metabolism biosynthesis, participate in drought tolerance related to the leaf permeability, by regulating the synthesis of flavonoids and cuticle.

Flavonoids represent a major component of the secondary metabolism in plants and comprise several major subgroups, including flavones, flavonols, anthocyanins, and proanthocyanidins. Flavonoids have been extensively described as defense metabolites for their role as antioxidants in protecting the plant from biotic and abiotic stresses by scavenging ROS [21]. MYB transcription factors in *Arabidopsis* have been demonstrated to be key regulators of flavonoid biosynthesis [22]. MYB12 upregulates the expression of the early flavonol biosynthetic genes *CHS* (*chalcone synthase*) and *FLS* (*flavonol synthase*), whereas the anthocyanin regulators MYB75/PAP1 control the expression of the late biosynthetic genes *DFR* (*dihydroflavonol reductase*) and *LDOX*/*ANS* (*leucoanthocyanidin dioxygenase*/*anthocyanidin synthase*) [23,24]. Overexpression of MYB12 and MYB75 in transgenic plants significantly increased the accumulation of flavonoids with strong antioxidant activity, thus leading to enhanced tolerance to abiotic stresses such as drought and oxidative stresses [22,25].

The cuticle is one of the most important plant structures and protects aerial plant organs from desiccation [26]. The main components of the cuticle contain cutin and wax, the biosynthesis of which is widely regulated by MYB transcription factors. In *Arabidopsis*, MYB41 negatively regulates the expression of cutin synthesis genes; MYB30, MYB94, and MYB96 function as positive regulators of wax synthesis genes, while MYB16 together with MYB106 can activate the synthesis of both cutin and wax [27]. Among these transcription factors, MYB41, MYB94, and MYB96 have been found to be involved in the drought response. Drought induction of cuticular wax biosynthetic genes requires the expression of *MYB94* and *MYB96*. MYB94 and MYB96 additively activate cuticular wax biosynthesis in response to drought by directly promoting the expression of the same wax biosynthetic genes. Overexpression of MYB96 leads to the upregulation of cuticular wax biosynthesis, thus contributing to drought tolerance in both *Arabidopsis* and *Camelina sativa*. Although the *myb94* mutant showed a phenotype similar to the wild type, the water loss rate of the *myb94 myb96* double mutant through the cuticle was faster, suggesting the additive role of MYB94 with MYB96 [28,29,30,31]. MYB41 negatively regulated the expression of the cutin synthesis genes *ATT1* and *LACS2*. Similarly to other cuticle biosynthesis mutants, the MYB4 overexpression plants exhibited a dwarf appearance, with smaller cells, and were characterized by an abnormal morphology. The expression of *MYB41* can be significantly induced in response to desiccation and ABA. Transgenic plants also show hypersensitivity to desiccation due to the enhanced permeability of leaf surfaces [32].

### 3.2. Light and ABA-Mediated Stomatal Movement

Plants use stomata on the epidermis to exchange carbon dioxide and water with the atmosphere. Regulation of the stomatal aperture is one of the most important ways for plants to control water loss [33]. *Arabidopsis* MYB transcription factors have been found to be widely involved in the drought response via stomatal movement. MYB60 is the first transcription factor shown to participate in the regulation of stomatal movement. *MYB60* is specifically expressed in guard cells, and its expression is induced by signals such as white and blue light that promote stomatal opening, but repressed by signals that promote stomatal closure, such as darkness, desiccation, and ABA treatment. Light-induced stomatal opening was repressed in the *myb60* mutant, thus enhancing plant drought tolerance via less water loss [34]. Conversely, MYB60 overexpression plants exhibited hypersensitivity to drought stress [35].

The other MYBs found so far mainly rely on ABA signals to participate in the regulation of plant stomatal movement. AtMYB44 is highly expressed in guard cells. However, its role in drought tolerance is controversial. Jung et al. [36] revealed that overexpression of MYB44 led to enhanced ABA sensitivity in both germination and stomatal closure, resulting in increased drought tolerance. Jaradat et al. [37] demonstrated that AtMYB44 negatively regulated ABA signaling. The involvement of MYB44 in ABA signaling seems to occur through the regulation of the ABA receptor RCARs/PYR1/PYLs. MYB44 can interact with RCAR3/PYL8 and RCAR1/PYL9 [37,38]. MYB44 competes with ABI1 to bind to RCAR1/PYL9, thus releasing the inhibitory effect of RCAR1/PYL9 on ABI1 phosphatase activity [38]. AtMYB77 can interact with MYB44 to form a heterodimer and additively function with MYB44 under drought stress. The *myb44 myb77* double mutants exhibited stronger drought tolerance [36]. MYB77 can also interact with RCAR3/PYL8 and RCAR1/PYL9 [37,38]. The PYL8–MYB77 interaction enhanced MYB77 activity to induce the expression of multiple auxin-responsive genes, thus promoting lateral root growth [39].

Despite wax synthesis, MYB96 also regulates drought resistance through the ABA signaling pathway. When expressed in leaves, MYB96 is localized mainly to guard cells. The stomatal aperture is more sensitive to ABA and drought in AtMYB96-overexpression plants but less sensitive in *myb96* null mutants. The *myb96* mutant showed a more susceptible phenotype in its drought response [40]. Lipid transfer protein 3 works as a target of MYB96 in ABA signaling to modulate MYB96-mediated drought tolerance. MYB96 positively regulates the expression of *LTP3* via direct binding to its promoter. The *ltp3* null mutant is less sensitive to drought stress, whereas overexpression of LTP3 can restore the hypersensitivity of the *myb96* mutant to drought stress [41]. MYB96 can also negatively regulate a subset of *ROP* genes, which act as negative regulators of multiple ABA responses, including stomatal closure and drought responses, via the histone modifier HDA15. *hda15* null mutants exhibited reduced ABA sensitivity and were more susceptible to drought stress than the *myb96* mutant. MYB96 can interact with HDA15 and recruit HDA15 to the promoters of *ROP6*, *ROP10*, and *ROP11*, thus repressing their expression by removing acetyl groups of histone H3 and H4 from the cognate regions [42].

*Arabidopsis* FLP (FOUR LIPS; MYB124) and its closest homolog MYB88 are critical for the formation of stomata. Stomata are developed from GMCs (guard mother cells), which divide symmetrically to produce two cells of equal size and fate. *FLP* is expressed late in the stomatal cell lineage to restrict cell divisions before terminal differentiation. FLP prevents new GMC daughter cells from perpetuating divisions, to restrict GMCs to a single division. In the *flp* mutant, GMCs undergo more than one round division, resulting in the formation of stomatal clusters. Mutations in MYB88 showed no stomatal phenotype, but *flp myb88* double mutants exhibited larger stomatal clusters than *flp* alone, indicating an additive role of MYB88 with FLP. As transcription factors, FLP and MYB88 can repress the expression of the mitosis-inducing factor *CDKB1;1*, which is specifically required for the last division in the stomatal pathway by directly binding to its promoter. However, the mutation of *cdkb1;1* can repress stomatal clusters of *flp myb88* double mutants [43,44]. FLP and MYB88 are also involved in ABA-mediated stomatal movement. ABA-induced stomatal closure is significantly repressed in *flp myb88* double mutants, resulting in the mutant having a phenotype more susceptible to drought stresses. Additionally, FLP and MYB88 control the drought stress response partially by regulating the expression of the NAC transcription factor, *NAC019*. The expression of *NAC019* is induced by drought, and overexpression of NAC019 can increase plant drought tolerance. FLP and MYB88 directly activate *NAC019* gene expression to promote the expression of downstream stress-responsive genes [45,46].

At the same time, there are still many other MYB proteins involved in the ABA-mediated stomata closure, but their functions are not yet clear. MYB20 functions as a negative regulator of ABA-mediated stomatal closure. The expression of *MYB20* is suppressed by desiccation and ABA treatment. Stomatal closure is insensitive to ABA in MYB20-overexpressing plants but sensitive in MYB20 knockout mutations. Thus, overexpression of MYB20 plants showed increased susceptibility to desiccation, and *myb20* null mutants exhibited enhanced resistance to desiccation [47]. MYB15 and MYB37 play a positive role in the plant response to ABA and drought stress. The expression of *MYB15* and *MYB37* can be strongly induced by ABA. Overexpression of MYB15 and MYB37 improves plant tolerance to drought by promoting ABA-induced stomatal closure [48,49].

## 4. MYBs in Regulation of ABA Signaling

ABA plays critical roles in plant responses to abiotic stresses and is also involved in multiple physiological processes, such as seed germination and dormancy, osmotic regulation, and leaf senescence [50]. In addition to ABA-mediated stomatal closure, many MYB transcription factors also participate in other ABA-regulated signaling pathways. For example, MYB15, MYB37, and MYB96 are also involved in ABA-mediated seed germination repression, while MYB77 and MYB96 modulate lateral root development in response to ABA [7,40,48,49]. Additionally, there are many MYB proteins that have been identified to be involved in the ABA signaling pathway. AtMYB2 participates in ABA-mediated seed germination through the regulation of the expression of the *RD22* and miRNA gene, *miR399f*. MYB2 positively regulates the expression of *RD22*, which is a dehydration-responsive gene induced by the application of exogenous ABA, by binding to the promoter of *RD22*. MYB2 is induced by drought and ABA treatment, and overexpression of MYB2 causes hypersensitivity to ABA during germination [51]. MYB2 also activates the expression of *miR399f* by directly binding to a MYB-binding motif in its promoter [52]. The expression of *miR399f* can also be induced by ABA treatment, and plants overexpressing miR399f exhibited a hyposensitive phenotype in response to ABA during seed germination and root growth but hypersensitivity to drought stress [53].

MYB33 and MYB101 have been identified as positive regulators of ABA signaling during germination. Null mutants of *myb33* and *myb101* showed hyposensitivity to ABA. However, their function in ABA signaling is still not clear. Meanwhile, MYB33 and MYB101 have been identified as targets of miR159 (microRNA 159). miR159 mediates the cleavage of MYB101 and MYB33 transcripts. Overexpression of miR159 can suppress the expression of *MYB33* and *MYB101*, thus rendering plants hyposensitive to ABA. Furthermore, ABA induced the accumulation of miR159 in germinating seeds, miR159-mediated MYB33, and MYB101 transcript degradation seems to act as a negative feedback regulation to fine-tune ABA signaling during seed germination [54]. Plants overexpressing MYB52 showed hypersensitivity to ABA during postgermination and root growth, and enhanced drought tolerance with higher survival rates under desiccation, suggesting that MYB52 is a positive regulator of ABA signaling [55].

In contrast, MYB7 and MYB30, which are involved in cuticle synthesis, act as negative regulators in ABA signaling. MYB7 negatively regulates ABA-induced inhibition of seed germination by blocking the expression of *ABI5*. Compared to the wild type, the *ABI5* expression is much higher in *myb7* mutant, and thus leading to hypersensitivity to ABA during seed germination [56]. MYB30 was first identified as the key positive regulator of HR (hypersensitive response) by modulating the synthesis of VLCFAs (very-long-chain fatty acids), the components of cuticular wax. MYB30 also belongs to the S1 subgroup with MYB96 and MYB94. However, unlike MYB96, MYB30 negatively regulates ABA signaling in seeds, as *myb30* null mutants exhibited hypersensitivity to ABA during seed germination and postgermination growth. However, the *myb30* mutant showed no differences compared to the wild type under drought stress. Considering that MYB30 can interact with MYB96 and their opposite roles in ABA signaling, MYB30 may participate in the regulation of ABA signaling through a complex regulatory network [40,57,58].

## 5. MYBs Modulate Salt Tolerance

**Figure 2:**
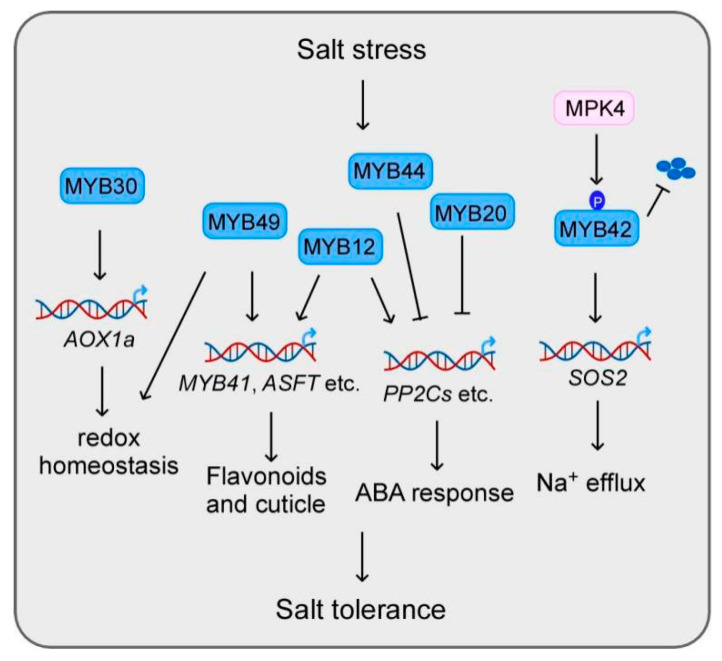
MYB transcription factors participate in salt stress through the regulation of downstream target genes. MYB44 and MYB20 positively regulate plant salt tolerance through the repression of key ABA repressor, *PP2Cs* [36,61]. MYB42 enhanced the expression of *SOS2* to facilitate Na^+^ efflux, while MPK4 phosphorylates MYB42 to protect it from degradation under salt stress [62]. MYB30 activates AOX1a expression to keep cellular redox homeostasis and MYB49 modulates cuticle formation and antioxidant defense [60,63]. Besides its role in flavonoids and cuticle formation, MYB12 can also induce the expression of ABA biosynthesis genes to confer plant salt tolerance [25].

Salt stress is one of the major environmental factors that limits plant growth and productivity. Salt stress can lead to ionic stress, osmotic stress, and secondary stresses, especially oxidative stress [59]. MYB proteins also contribute to plant salt tolerance and many of their downstream targets have been identified (Figure 2). MYB49 positively modulates salt tolerance by modulating cuticle formation and antioxidant defense. The null mutant of MYB49 displayed higher electrolyte leakage and less tolerance to salt stress, while overexpression of MYB49 improved salt tolerance. MYB49 was found to activate the expression of cutin, suberin, and wax biosynthetic genes such as *MYB41*, *ASFT*, *FACT*, and *CYP86B1* by directly binding to their promoters. Meanwhile, MYB49 can elevate Ca^2+^ levels in leaves and improve antioxidant capacity by upregulating genes encoding peroxidases and late embryogenesis abundant proteins [60].

Many MYB proteins involved in the ABA response also participate in regulating salt tolerance through the regulation of genes in ABA signaling. The expression of *MYB20* can be induced by NaCl treatment, and overexpression of MYB20 enhanced salt stress tolerance. MYB20 negatively regulates the expression of *PP2Cs*, the major negative regulators of ABA signaling, by directly binding to their promoters, thus leading to salt tolerance in plants [61]. MYB44 also participates in the salt response through the regulation of PP2Cs. Salt-induced activation of PP2C genes, such as *ABI1*, *ABI2*, *AtPP2CA*, *HAB1*, and *HAB2* was diminished in MYB44-overexpressing plants, but enhanced in the *myb44* mutant. Overexpression of MYB44 can enhance tolerance to salt stress [36]. MYB7 participates in salt stress during seed germination though the negative regulation of ABI5. Mutation of MYB7 can cause hypersensitivity to salt stress during seed germination [56].

MYB42 positively modulates salt tolerance through the regulation of *SOS2* (*salt overly sensitive 2*) expression. SOS2 encodes a serine/threonine protein kinase and activates Na^+^/H^+^ exchanger SOS1 through phosphorylation, thus facilitating Na^+^ efflux for enhancing plant salt tolerance. The *myb42* mutant exhibited enhanced salt sensitivity, while MYB42-overexpression lines improved salt tolerance compared with the wild type. MYB42 activates the expression of *SOS2* gene by directly binding to *SOS2* promoter, thus inducing its expression under salt stress. MPK4 (mitogen-activated protein kinase 4) participates in salt tolerance through the regulation of MYB42. NaCl treatment can enhance the interaction of MPK4 with MYB42, thus leading to the phosphorylation of MYB42. Salt treatment can induce the degradation of MYB42. This phosphorylation enhances the stability of MYB42 under salt-stress conditions [62].

MYB30 modulates plant salt tolerance through the regulation of mitochondrial alternative oxidase AOX1a (alternative oxidase 1a). MYB30 mutants exhibited hypersensitivity to salt stress. In response to salt stress, MYB30 directly binds to the promoter of *AOX1a* to promote its expression, thus maintaining the cellular redox homeostasis through enhanced Alt respiration. Overexpression of AOX1a can rescue the *myb30* salt-sensitive phenotype [63]. In addition to flavonoid biosynthesis and ROS scavenging, overexpression of MYB12 also upregulated the expression of genes involved in ABA and proline biosynthesis, thus conferring salt tolerance to the plants [22,25].

Besides the targets of MYB proteins, how MYBs are regulated in salt stress is revealed by the study of MYB74. MYB74 plays a positive role in the salt response. NaCl treatment can induce the expression of *MYB74.* Transgenic plants overexpressing MYB74 displayed hypersensitivity to NaCl during seed germination. Further analysis showed that the expression of *MYB74* in response to salt stress is under the control of the RdDM (RNA-directed DNA methylation) pathway. Twenty-four-nt siRNAs target a region approximately 500 bp upstream of the transcription initiation site of MYB74. This target site for siRNA is essential for maintaining *MYB74* expression patterns and has been shown to be heavily methylated via the RdDM pathway. Salt stress induces the expression of *MYB74* by repressing the accumulation of 24-nt siRNAs and the RdDM pathway [64].

In addition to the above genes, many other MYB proteins are also involved in the salt tolerance response. For example, transgenic plants overexpressing MYB41 showed enhanced salt tolerance on germination and root growth. MYB15 overexpression lines displayed improved salt stress tolerance [48]. *myb108* and *flp myb88* mutant plants were significantly more susceptible to high salt during seed germination [45,65]. In contrast, MYB52 negatively regulates plant salt tolerance, and overexpression of MYB52 causes hypersensitivity to salt stress on seed germination [55]. However, their functions in salt stress still need to be investigated.

## 6. MYBs Serve as Repressors in Cold and Heat Tolerance

Cold stress threatens the growth and development of plants by greatly affecting plant metabolism and transcriptomes. Cold stress rapidly induces the expression of *CBFs* (*C-repeat-binding factors*), the key transcription factors in the cold response, in order to activate the expression of numerous downstream *COR* (*cold-responsive*) genes [66]. MYB transcription factors are considered to play critical roles in cold tolerance because of their regulation of *CBF* genes. Among them, AtMYB15 has been identified as the key regulator of *CBF* genes in response to low temperature. The expression of *MYB15* can be induced by cold stress. The *myb15* mutant plants showed increased tolerance to freezing stress, whereas the overexpression plants exhibited reduced freezing tolerance. MYB15 repressed the expression of *CBF* genes by directly binding to MYB binding sites in their promoters. Cold-induced *CBF* expression was reduced in MYB15-overexpressing plants but enhanced in the *myb15* mutant. At the same time, MYB15 can interact with another key CBF regulator, ICE1, to modulate the expression of *CBF* genes, indicating the multiple roles of MYB15 in controlling the expression of *CBFs* during cold stress [67]. The activity and stability of MYB15 are precisely controlled by posttranslational modifications. MPK6 interacts and phosphorylates MYB15 at Ser-168. MPK6-induced phosphorylation reduced the affinity of MYB15 binding to the *CBF3* promoter. Compared with MYB15-overexpressing plants, overexpression of MYB15^S168A^ caused reduced *CBF* transcript levels in response to cold stress and enhanced sensitivity to freezing [68]. Cold induces the U-box type E3 ubiquitin protein PUB25- and PUB26-mediated degradation of MYB15 to transport cold signaling. PUB25 and PUB26 interact with MYB15 and mediate the ubiquitination of MYB15. The freezing sensitivity in the *pub25 pub26* double mutants can largely be rescued by the MYB15 mutation [69].

MYB96 is also induced by cold stress and subsequently activates freezing tolerance through the regulation of *CBF* expression. Cold induction of *CBF* and *COR* genes is diminished in the *myb96* mutant. MYB96 regulates the expression of *CBFs* through HHP proteins. MYB96 positively regulates the expression of *HHP1*, *HHP2*, and *HHP3* by directly binding to their promoters. HHP1 proteins in turn positively regulate the transcriptional activity of ICE1 through protein–protein interactions and thereby activate downstream *CBF* expression. The enhanced cold tolerance of the *myb96* mutant can be repressed by the mutation of *HHP* genes, suggesting that MYB96 acts as an upstream regulator of the cold response [70]. Additionally, MYB96 also participates in the cold response through the regulation of LTP3. Overexpression of LTP3 enhanced plant cold tolerance and recovered the hypersensitivity of the *myb96* mutant to cold stress [41].

High-temperature stress can also cause damage to the physiological metabolism and growth of plants. MYB30 participates in oxidative and heat stress responses through the regulation of calcium signaling. Plants lacking MYB30 protein exhibit enhanced elevation of [Ca^2+^]_cyt_ (free Ca^2+^) in response to H_2_O_2_ and heat stimuli. *myb30* null mutants exhibited more sensitivity to oxidative stress but resistance to heat shock treatment. The MV and heat sensitivity of *myb30* can be suppressed by the application of LaCl_3_, a calcium channel blocker that blocks [Ca^2+^]_cyt_ elevation. MYB30 regulates calcium signaling by repressing the *ANN* (*ANNEXIN*) genes, which encode membrane Ca^2+^ transporter proteins modulating cytosolic calcium signatures. MYB30 can directly bind to the promoters of *ANN1* and *ANN4* to repress their expression. ANN mutations not only blocked abnormal [Ca^2+^]_cyt_ in the *myb30* mutant induced by H_2_O_2_ and heat treatment but also suppressed the MV and heat sensitivity of the *myb30* mutant [71].

## 7. Posttranslational Modifications

The transcriptional activity and protein stability of MYB proteins are precisely regulated by multiple posttranslational modifications. MPK-mediated phosphorylation seems to be one of the major regulators of the activity and stability of MYB proteins. Besides MYB15, MPK6 phosphorylated MYB41 at Ser-251 and enhanced its DNA binding to the promoter of an *LTP* gene. Overexpression of MYB41 wild-type protein, but not the mutated protein, can enhance plant salt tolerance [72]. MPK4 participates in light-induced anthocyanin accumulation through the regulation of MYB75. MPK4 interacts with MYB75 and mediates the phosphorylation of MYB75. Phosphorylation of MYB75 can increase its stability. Thus, MPK4 promotes anthocyanin biosynthesis through the maintenance of MYB75 stability [73]. In addition to MPK4, MPK3, MPK6, and MPK11 can also interact with MYB75 and mediate its phosphorylation, suggesting the important role of the MPK-MYB75 signaling cascade in anthocyanin biosynthesis [74].

The ubiquitin/26S proteasome pathway is responsible for selective protein turnover. Ubiquitin E3 ligases catalyze the covalent addition of ubiquitin to the target protein and define substrate specificity [75]. Many RING-type E3 ligases have been found to mediate the degradation of MYB proteins. The RING-type ubiquitin E3 ligase COP1 (constitutively photomorphogenic1) mediates light-induced anthocyanin biosynthesis by regulating the stability of MYB75 and MYB90/PAP2. COP1 can interact with MYB75 and MYB90. MYB75 and MYB90 proteins are degraded in the dark, and degradation is dependent on COP1 [76].

The RING-type E3 ubiquitin ligase MIEL1 (MYB30-interacting E3 ligase 1) participates in the HR response by regulating the stability of MYB30. MIEL1 interacts with and ubiquitinates MYB30, leading to MYB30 proteasomal degradation and downregulation of its transcriptional activity. Mutation of MYB30 can restore the hypersensitive phenotype of the *miel1* mutant to the HR response [77]. MIEL1 is also involved in ABA regulation of seed germination by promoting MYB96 turnover. MIEL1 can interact with MYB96 and stimulate its ubiquitination and degradation. In contrast to MYB96, the MIEL1-deficient mutant displayed hypersensitivity to ABA during seed germination, whereas MIEL1-overexpressing plants were less sensitive. The seed germination rate of the *miel1 myb96* double mutant is similar to that of *myb96* under ABA treatment, suggesting that MYB96 is epistatic to MIEL1 in the control of seed germination [78].

The degradation of MYB30 in ABA signaling is modulated by the RING-H2 E3 ligase RHA2b. RHA2b interacts with MYB30 to ubiquitinate MYB30, mainly at the Lys-283 site. RHA2b positively regulates the ABA response, as the *rha2b* mutant is hypersensitive to ABA during seed germination. The stability of MYB30 is significantly improved in the *rha2b* mutant. MYB30 mutation can block the hyposensitive phenotype of the *rha2b* mutant to ABA, suggesting that RHA2b participates in seed germination by controlling the stability of MYB30 [79]. In contrast, the stability of MYB30 is maintained by the modification of SUMOylation. The mechanism of SUMOylation is similar to the mechanism of ubiquitination. SUMOE3 ligases catalyze the covalent addition of small ubiquitin-like modifier (SUMO) proteins to the target protein. The SUMO E3 ligase SIZ1 participates in ABA signaling by protecting MYB30 from degradation through SUMOylation. SIZ1-mediated SUMOylation plays critical roles in maintaining the stability and transcriptional function of MBY30. Additionally, the SUMOylation site is also localized at the Lys-283 site, suggesting that SUMOylation can protect MYB30 from ubiquitin-mediated proteasomal degradation by occupying the same modification site [58,79]. While SUMO proteases mediate the reversible process of SUMOylation, the deconjugation of SUMO forms the target proteins. ASP1 (SUMO protease 1) positively regulates ABA signaling during early seed development through the regulation of MYB30. In the *asp1* mutant, the stability of MBY30 is enhanced, and ABA can induce the expression of *ASP1* to modulate the stability of MYB30 [80].

## 8. Conclusions and Outlook

Multiple studies have shown that MYB proteins not only participate in the biosynthesis of metabolites such as flavonoids and cuticles, but also play critical roles in the response to abiotic stresses, including drought and cold stresses (Table 1). However, how these MYB proteins are regulated during these processes is still not very clear. Although the expression of *MYB* genes can be induced by various signals, little is known about the underlying mechanism. For example, MYB4 can inhibit the expression of its own genes, and RdDM-mediated DNA methylation regulates the expression of *MYB74*. Another question is how to regulate the activity and stability of MYB proteins at the posttranslational level. Phosphorylation and ubiquitination have been shown to play important roles. MPK kinases seem to serve as the major kinases that mediate the phosphorylation of MYB proteins. MPK6 participates in anthocyanin biosynthesis and salt and cold tolerance through the phosphorylation of MYB75, MYB41, and MYB15, respectively. Ubiquitination and SUMOylation antagonistically regulate the stability of the MYB30 protein to modulate ABA signaling in the seed germination process. These findings provide clues for understanding how MYB proteins are regulated at the protein level. Additionally, the MBW transcription factor complex plays important roles in flavonoid pigment biosynthesis, indicating that MYB proteins can function together with other transcription factors. For example, BES1, a key transcription factor in BR signaling, can interact with MYB30, thus activating the expression of BR-responsive genes together with MYB30 [81]. At the same time, MYB30 can interact with MYB96. Although they are both involved in ABA signaling, MYB30 and MYB96 have opposite functions in ABA-mediated seed germination inhibition, suggesting a complicated mechanism for the coregulation of transcription factors. Nevertheless, the function of MYB proteins is in need of much more investigation in the future.

## Figures and Tables

**Table 1:** MYB genes involved in abiotic stress responses.

Gene Name	Abiotic Stresses Involved	Function Description	References
MYB4	UV-B tolerance	Negatively regulates the expression of *C4H*	[12]
MYB7	UV-B, ABA, salt stress	Negatively regulates flavonols biosynthesis and *ABI5* expression	[14,56]
MYB73 MYB77	UV-B	Function together to positively regulate the expression of auxin-responsive genes, interact with UVR8	[19]
MYB13	UV-B	Regulates the expression of auxin-responsive and flavonoid biosynthetic genes, interact with UVR8	[20]
MYB12	Drought, oxidative stresses	Positively regulates flavonol biosynthetic genes, *CHS* and *FLS*	[22,23,25]
MYB75	Drought, oxidative stresses	Positively regulates flavonol biosynthetic genes, *DFR* and *LDOX*	[22,24]
MYB41	Drought, salt stresses	Negatively regulates cutin synthesis genes, *ATT1* and *LACS2*	[32]
MYB60	Drought stress	Specifically expressed in guard cells, promotes stomata closure	[34]
MYB30	ABA, salt, heat, oxidative stresses	Modulates the synthesis of VLCFAs, positively regulates the expression of *AOX1a*, represses *ANNs* expression	[57,58,63,71]
MYB96	ABA, drought, cold stresses	Activates cuticular wax biosynthesis, positively regulates the expression of *HHPs* and *LTP3*	[28,40,41,70]
MYB94	Drought stress	Additively activates cuticular wax biosynthesis with MYB96	[30,31]
MYB88 MYB124	Drought stress	Prevents the division of GMC daughter cells, activates *NAC019* gene expression, repress the expression of *CDKB1;1*	[44,45,46]
MYB2	ABA	Positively regulates the expression of *RD22* and *miR399f*	[51,52]
MYB33 MYB101	ABA	Targets of miR159	[54]
MYB20	Drought, salt stresses	Negatively regulates the expression of *PP2Cs*	[47,61]
MYB49	Salt stress	Modulates the cuticle formation and antioxidant defence	[60]
MYB42	Salt stress	Positively regulates the expression of *SOS2*	[62]
MYB74	Salt stress	Regulated by 24-nt siRNAs and RdDM pathways	[64]
MYB15	ABA, drought, salt, cold stresses	Key repressor of cold response, represses the expression of *CBF* genes, interacts with ICE1	[48,67]
MYB108	Salt stress	Unknown	[65]
MYB37	ABA, drought stress	Unknown	[49]
MYB52	ABA, salt stress	Unknown	[55]

## Data Availability

Not applicable.
